# Metabolites Profiling of Melanoma Interstitial Fluids Reveals Uridine Diphosphate as Potent Immune Modulator Capable of Limiting Tumor Growth

**DOI:** 10.3389/fcell.2021.730726

**Published:** 2021-09-17

**Authors:** Eleonora Vecchio, Carmen Caiazza, Selena Mimmi, Angelica Avagliano, Enrico Iaccino, Teresa Brusco, Nancy Nisticò, Domenico Maisano, Annamaria Aloisio, Ileana Quinto, Maurizio Renna, Giuseppina Divisato, Simona Romano, Martina Tufano, Massimo D’Agostino, Elena Vigliar, Antonino Iaccarino, Chiara Mignogna, Francesco Andreozzi, Gaia Chiara Mannino, Rosangela Spiga, Mariano Stornaiuolo, Alessandro Arcucci, Massimo Mallardo, Giuseppe Fiume

**Affiliations:** ^1^Department of Experimental and Clinical Medicine, University of Catanzaro “Magna Graecia”, Catanzaro, Italy; ^2^Department of Molecular Medicine and Medical Biotechnology, University of Naples Federico II, Naples, Italy; ^3^Department of Public Health, University of Naples Federico II, Naples, Italy; ^4^Department of Health Sciences, Magna Graecia University, Catanzaro, Italy; ^5^Department of Medical and Surgical Sciences, University “Magna Graecia” of Catanzaro, Catanzaro, Italy; ^6^Department of Pharmacy, University of Napoli Federico II, Naples, Italy

**Keywords:** metabolite profiling, TIL (tumor infiltrating lymphocytes), anti-tumor immunity, B16 engraftment, UDP

## Abstract

Tumor interstitial fluid (TIF) surrounds and perfuses tumors and collects ions, metabolites, proteins, and extracellular vesicles secreted by tumor and stromal cells. Specific metabolites, accumulated within the TIF, could induce metabolic alterations of immune cells and shape the tumor microenvironment. We deployed a metabolomic approach to analyze the composition of melanoma TIF and compared it to the plasma of C57BL6 mice, engrafted or not with B16-melanoma cells. Among the classes of metabolites analyzed, monophosphate and diphosphate nucleotides resulted enriched in TIF compared to plasma samples. The analysis of the effects exerted by guanosine diphosphate (GDP) and uridine diphosphate (UDP) on immune response revealed that GDP and UDP increased the percentage of CD4^+^CD25^+^FoxP3^–^ and, on isolated CD4^+^ T-cells, induced the phosphorylation of ERK, STAT1, and STAT3; increased the activity of NF-κB subunits p65, p50, RelB, and p52; increased the expression of Th1/Th17 markers including IFNγ, IL17, T-bet, and RORγt; and reduced the expression of IL13, a Th2 marker. Finally, we observed that local administrations of UDP in B16-engrafted C57BL6 mice reduced tumor growth and necrotic areas. In addition, UDP-treated tumors showed a higher presence of MHCII^hi^ tumor-associated macrophage (TAM) and of CD3^+^CD8^+^ and CD3^+^CD4^+^ tumor-infiltrating T-lymphocytes (TILs), both markers of anti-tumor immune response. Consistent with this, intra-tumoral gene expression analysis revealed in UDP-treated tumors an increase in the expression of genes functionally linked to anti-tumor immune response. Our analysis revealed an important metabolite acting as mediator of immune response, which could potentially represent an additional tool to be used as an adjuvant in cancer immunotherapy.

## Introduction

Melanoma tumor is constituted by cancer cells and non-cancerous cells including immune cells, mesenchymal stem cells, niche cells, cancer-associated fibroblasts, and adipocytes as well as the blood and lymphatic vascular systems, the extracellular matrix (ECM), and signaling molecules, which define the tumor microenvironment (Wang et al., 2017). Cancer cells are able to modulate such a complex ecosystem through the secretion of cytokines, chemokines, and other signaling molecules, which in turn sustain and promote the tumor growth (Prager et al., 2019). Several types of immune cells are involved in tumor immuno-surveillance, and among those, a critical role is played by the tumor-infiltrating T-lymphocytes (TILs). The immune system, through the action of different cells including T-lymphocytes, natural killer (NK) cells, and macrophages, provides an essential protection, which is instrumental for the detection and eradication of pre-malignant and cancerous cells. However, the altered metabolism of cancer cells influences the nutrient composition of the tumor microenvironment (e.g., glucose, lactate, Krebs cycle metabolites, amino acids) and as a consequence can modulate the anti-tumoral activity of immune cells (Lyssiotis and Kimmelman, 2017; Avagliano et al., 2020a, b). Therefore, by altering the metabolic status of immune cells, tumors are able to elicit immune dysfunctions, including the aberrant production of cytokines, altered cytotoxicity, and macrophages polarization toward pro-tumoral phenotypes (Ho et al., 2015; Gonzalez et al., 2018).

Tumor interstitial fluid (TIF) surrounds and perfuses tumors and collects ions, metabolites, extracellular proteins, and vesicles either secreted by tumor and stromal cells or derived from tumor necrotic areas. In this context, the accumulation of specific metabolites within the interstitial fluid could induce metabolic alterations of immune cells that could undertake specific differentiation pathways and produce a “cytokine storm,” ultimately supporting a pro-tumor microenvironment (Wagner and Wiig, 2015; Gromov and Gromova, 2016; Sullivan et al., 2019). Hence, the analysis of TIF composition can certainly represent a powerful tool for the identification of novel cancer biomarkers and could potentially provide novel therapeutic strategies in cancer treatments.

Here, by using an omics approach, we have investigated and compared the metabolomic profiles of melanoma TIF to plasma derived from C57BL6 mice, engrafted or not with the well-established B16 melanoma cell line (Bohm et al., 1998; Tuccillo et al., 2014). Next, we evaluated the effects on activation markers of the immune response of selected metabolites, including the guanosine diphosphate (GDP) and uridine diphosphate (UDP) nucleotides, whose presence resulted in change as a function of the engraftment. Strikingly, we observed that in C57BL6 mice engrafted with B16 melanoma cells, local injection of UDP reduced tumor growth and necrosis by recruiting immune infiltrates, which express anti-tumor immunity markers. Our analysis revealed UDP as an important metabolite acting as a key mediator of immune response, which could potentially represent a valuable tool to be used as an adjuvant in cancer immunotherapy.

## Methods

### Cell Cultures and Treatments

B16F10 tumor cell lines were purchased from the American Type Culture Collection, Manassas, VA, United States. B16F10 were cultured in Dulbecco’s modified Eagle’s medium supplemented (DMEM, Life Technologies, Paisley, United Kingdom) with 10% heat-inactivated fetal calf serum (Life Technologies, Paisley, United Kingdom) and 2 mM L-glutamine (Lonza Cologne AG, Germany).

Human peripheral blood mononuclear cells (PBMCs) were derived from buffy coats of healthy donors and isolated by Ficoll Paque gradient (GE Healthcare Europe, Munich, Germany), as previously described (Fiume et al., 2013; Pontoriero et al., 2019). Briefly, blood samples were diluted 1:1 in phosphate-buffered saline (PBS) and stratified on Ficoll solution with a 3:1 (v/v) ratio. After 30 min centrifugation at 400 × *g*, PBMCs were recovered and re-suspended in RPMI-1640 (Life Technologies, Paisley, United Kingdom) medium supplemented with 10% fetal calf serum. CD4^+^ or CD8^+^ T-cells were isolated from PBMCs by using CD4^+^ T Cell Isolation Kit or CD8^+^ T Cell Isolation Kit, respectively (Miltenyi Biotec, Germany).

PBMCs were treated with adenosine monophosphate (AMP), GDP and UDP (Sigma-Aldrich, St Louis, MO, United States) at a concentration of 30 μM for the indicated time, washed twice with PBS (Gibco, Life Technologies), and lysed in radioimmunoprecipitation assay (RIPA) buffer or in Trizol reagent (Life Technologies, Grand Island, NY, United States), to obtain protein extracts or total RNA extracts, respectively.

### Mice

Wild type C57BL6/J mice were obtained from The Jackson Laboratory (Bar Harbor, Maine; United States). For the B16F10 engraftment experiments in mice, B16F10 melanoma cells were grown in DMEM, detached, and resuspended in PBS (Gibco). Next, 2.0 × 10^5^ tumor cells were injected subcutaneously in the lateral flanks of 2-month-old female C57BL6/J mice. Twenty-one days post-engraftment, mice were sacrificed, and tumors were collected. It is worthy to note that B16 engrafted mice were monitored daily for signs of morbidity and tumor development, and moribund mice were sacrificed.

Experiments of UDP treatments of B16-melanoma-bearing mice were performed by injecting 100 μl of UDP (0.5 mM), in the same localization of B16-tumor engraftment, at days 3, 10, and 17 post-engraftments. The volume of the tumors was calculated by the following formula: Length × Width × Height and expressed in mm^3^.

### Isolation of Tumor Interstitial Fluid

Melanoma interstitial fluids were collected by using the procedure of Haslene-Hox (Haslene-Hox et al., 2011). Briefly, tumors were washed twice with 2 ml of PBS and then placed on a fine mesh and subjected to low-speed centrifugation at 10^6^ × *g* for 10 min. Fluid without erythrocytes were collected and stored at −80°C until further processing. Next, TIF samples were used for metabolomic analysis or for the dosage of specific cytokines and chemokines.

### Metabolomic Analysis of Tumor Interstitial Fluid and Plasma Samples by Liquid Chromatography-Mass Spectrometry

Blood and TIF samples were diluted with an equal volume of a 1:1 prechilled MetOH:H_2_O solution. The samples were vortex-mixed, kept on ice for 20 min, and centrifuged again at 10,000 × *g*, at 4°C for 10 min. The collected supernatant was dried up in SpeedVac concentrator system (Thermo Scientific), operated at room temperature. Dried supernatants were reconstituted with 125 μl of methanol/acetonitrile/water (50:25:25). Extracted metabolites were analyzed using an ACQUITY UPLC (ultra-performance liquid chromatography) system online coupled to a Synapt G2-Si QTOF-MS (Waters Corporation, Milford, MA, United States) in positive and negative modes in the following settings: reverse-phase ACQUITY UPLC CSH C18 (1.7 μm, 100 × 2.1 mm^2^) column (Waters), 0.3 ml/min flow rate, mobile phases composed of acetonitrile/H_2_O (60:40) containing 0.1% formic acid and 10 mM ammonium formate (Phase A), and isopropanol/acetonitrile (90:10) containing 0.1% formic acid and 10 mM ammonium formate (Phase B). Peak detection, metabolite identification, and quantitation were performed as previously described (10.3390/nu11010163), fitting experimental data with internal standard and calibration curves. Data analysis and heatmap were generated with the on-line software MetaboAnalyst,^1^ as previously reported (Riccio et al., 2018; Badolati et al., 2020).

### Analysis of Cytokines and Chemokines Expression Within Tumor Interstitial Fluid and Plasma Samples

Plasma samples were obtained from the blood of 2-month-old female mice, engrafted or not with B16-melanoma cells, by centrifuging at 800 × *g* for 10 min, while TIF samples were obtained as detailed above. Plasma or TIF aliquots (30 μl) were immediately assayed for cytokines and chemokines expression by using Mouse Multi-Analyte ELISArray Kits (Qiagen, Hilden, Germany), according to the manufacturer’s conditions.

### Histological Analysis by Hematoxylin and Eosin Staining

Histological analysis was performed as previously described (Granato et al., 2017). Briefly, B16-deriving melanoma tumors were wholly removed at necropsy, fixed in buffered formalin (10%), dehydrated, and paraffin embedded. The preparation of paraffin-embedded tumor sections was performed by using Shandon Cytoblock Cell Block Preparation System (Thermo Scientific, Rockford, IL, United States) according to the manufacturer’s protocol. Thick sections (5 μm) of each paraffin block were stained by hematoxylin and eosin (H&E) and then were converted into high-resolution digital data through NanoZoomer-2.0RS digital scanner (Hamamatsu, Tokyo, Japan, Asia). Images were taken in bright field with a digital camera (Leica DC200; Leica Microsystems) connected to the microscope. For the evaluation of necrotic areas, necrosis is defined as acellular regions (appearing pale violet) within tumors as identified by H&E stain, while cellular regions are characterized by well-defined boundaries. Regions of interest were manually traced and measured using the freehand tool in ImageJ.^2^

### Cells Extracts, Western Blotting, and NF-κB DNA Binding Assays

Total cell extracts were prepared as previously described (Schiavone et al., 2012). Briefly, cells were washed twice with PBS 1X and lysed in RIPA buffer [1% NP-40, 10 mM Tris-HCl, 150 mM NaCl, and 1 mM ethylenediamine tetraacetic acid (EDTA)], supplemented with a protease inhibitor cocktail (cOmplete-mini EDTA-free tablets—Roche) on ice for 20 min. Lysates were centrifuged for 10 min at 14,000 × *g*, 4°C. Protein extract aliquots were resolved on 12% sodium dodecyl sulfate polyacrylamide gel electrophoresis (SDS–PAGE), transferred to polyvinylidene difluoride membrane (PVDF, Millipore, Bedford, MA, United States), and incubated with primary antibodies (1:1,000) followed by incubation with secondary horseradish peroxidase (HRP)-conjugated antibodies (1:2,000) (GE Healthcare Amersham, Little Chalfont, United Kingdom) in PBS containing 5% non-fat dry milk (Bio-Rad Laboratories). Primary antibodies were purchased from Cell Signaling Technology (Phospho-p44/42 MAPK (Erk1/2) (Thr202/Tyr204) #4370, p44/42 MAPK (Erk1/2) Antibody #9102, Phospho-Stat3 (Tyr705) #9145, Stat3 Mouse mAb #9139, Phospho-Stat5 (Tyr694) Rabbit mAb #9359, Santacruz Biotechnology (p-Stat1 (pY701.4A sc-136229), and Thermo Fisher (STAT1 Monoclonal Antibody # AHO0832).

For the NF-kB binding activity assays, nuclear extracts were obtained as previously described (Janda et al., 2011; Pontoriero et al., 2019). Briefly, PBMCs were washed twice in PBS and resuspended in lysis buffer containing 10 mM 4-2-hydroxyethyl-1-piperazineethanesulfonic acid (HEPES) pH 7.9, 1.5 mM MgCl_2_, 10 mM KCl, 0.5 mM dithiothreitol (DTT), and 0.1% NP-40. Cells were lysed on ice for 2 min and checked for complete lysis by phase contrast microscopy. Nuclei were centrifuged at 800 × *g* for 5 min, and the supernatant was collected as cytosolic extracts. The nuclear pellet was washed with a buffer containing 10 mM HEPES pH 7.9, 1.5 mM MgCl_2_, 10 mM KCl, and 0.5 mM DTT and lysed in a buffer containing 20 mM HEPES pH 7.9, 25% glycerol, 0.45 M NaCl, 1.5 mM MgCl2, 0.2 mM EDTA, 0.5 mM DTT, 1 mM Phenylmethylsulfonyl fluoride (PMSF), and 1 × Complete Protease Inhibitor. Nuclear lysates were centrifuged at 14,000 × *g* for 15 min, and the supernatants were collected as nuclear extracts. The binding of the NF-κB subunits to the double-stranded NF-κB oligonucleotide probes was measured in nuclear extracts, using the NF-κB Transcription Factor Assay kit (Cayman Chemical Company, Ann Arbor, MI, United States), as previously described (Fiume et al., 2013; Pontoriero et al., 2019).

### Flow Cytometry

Flow cytometry experiments were performed using the antibodies anti-human CD4-FITC (VIT4), anti-human CD25-PE, anti-human/mouse FoxP3-APC, anti-mouse F4/80-PerCP (REA126), and anti-mouse MHCII-FITC (REA813) from Miltenyi Biotec, Bergisch Gladbach, Germany; the antibodies anti-mouse CD3-PerCP, anti-mouse CD4-PE, anti-mouse CD8-FITC, anti-mouse CD11b-APC, IgG2a-APC rat isotype, IgG2a-FITC, and IgG2a-PE rat isotype (R35–95) purchased from BD Biosciences; and the antibody anti-human CD8-APC from ImmunoTools GmbH, Germany. Briefly, PBMCs (1 × 10^6^ cells) were incubated with fluorochrome-conjugated antibodies (0.5 μg) for 30 min on ice. For the detection of T-regs, PBMCs (1 × 10^6^ cells) were incubated with anti-CD4-FITC (0.5 μg) and anti-CD25-PE (0.5 μg), followed by intracellular staining with anti-FoxP3-APC antibody (0.5 μg) using FoxP3 staining buffer (eBioscience^TM^ Intracellular Fixation and Permeabilization Buffer Set; Thermo Fisher Scientific, Monza, Italy). For the analysis of tumor immune infiltrates, melanoma tumors from B16-bearing mice treated with or without UDP were digested with 0.5 U/ml collagenase (Sigma-Aldrich, Milan, Italy), and cell suspensions were passed through 70-μm cell strainers and then stained with fluorochrome-conjugated antibodies (0.5 μg) for 30 min on ice. After two PBS washes, cells were analyzed by flow cytometry using BD FACSAria flow cytometer and BD FACSDiva software (BD Biosciences), as previously described (Janda et al., 2011; Fiume et al., 2015).

For the experiments of CD8^+^ T-cells proliferation, PBMCs 3 × 10^6^ were treated with CellTrace CFSE (carboxyfluorescein succinimidyl ester) (cell proliferation kit, Thermo Fisher Scientific) at a final concentration of 5 μm for 20 min at 37°C, according to the manufacturer’s protocol. Three days after CFSE labeling, CD8^+^ T-cells proliferation was measured, on gated CD8^+^ T-cells, by evaluating the cell percentage of fluorescence peaks related to daughter cell generations.

### Real-Time PCR

PBMCs were treated with AMP, GDP, or UDP or left untreated for 12 h and then washed by addition of cold PBS. Next, CD4^+^ or CD8^+^ T-cells were isolated by using CD4^+^ or CD8^+^ isolation kit (Miltenyi Biotec), and total RNA was extracted from cells using the TRIzol reagent (Invitrogen). Equal amounts of total RNA (200 ng) were reverse transcribed using the Random Hexamers (Roche) and SuperScript III Reverse Transcriptase according to the manufacturer’s protocol (Invitrogen). Real-time quantitative PCR (RT-qPCR) was performed with the iQ Green Supermix (Bio-Rad Laboratories) and carried out with the iCycler iQ Real-Time Detection System (Bio-Rad Laboratories) under the following conditions: 95°C, 1 min; (94°C, 10 s; 60°C, 30 s) × 40. RT-qPCR of immune markers was performed using the RT2 profiler PCR Array-Human NF-κB signaling pathway (QIAGEN Sciences, MD, United States) and by using a set of specific primers, listed in Supplementary Materials. For the analysis of transcripts encoding for immune markers within tumors deriving from B16-melanoma-bearing mice, total RNA was extracted from tumor cell suspensions (5 × 10^6^) and reverse transcribed as previously described (Vitagliano et al., 2011; Albano et al., 2018). RT-qPCR of immune markers was performed using the RT2 profiler PCR Array-Mouse B&T cell activation markers (QIAGEN). Reactions were carried out in triplicate, and gene expression levels were calculated relative to glyceraldehyde 3-phosphate dehydrogenase (GAPDH) mRNA levels as endogenous control. Relative expression was calculated as 2 (Ct gene under investigation - Ct GAPDH).

### Statistical Analysis

Statistical analysis was performed by the two-tailed unpaired Student’s *t-*test. Data were reported as mean values ± SE. Differences between the means were accepted as statistically significant at the 99% level (*p* < 0.01).

## Results

### Isolation and Characterization of Melanoma Tumor Interstitial Fluid

In our work, we asked whether metabolites released in the tumor microenvironment by tumor or stromal cells could modulate the immune response. To this aim, we deployed a metabolomic approach to analyze the composition of melanoma TIF and compare it to plasma of C57BL6 mice, engrafted or not engrafted with B16 melanoma cells.

**FIGURE 1:**
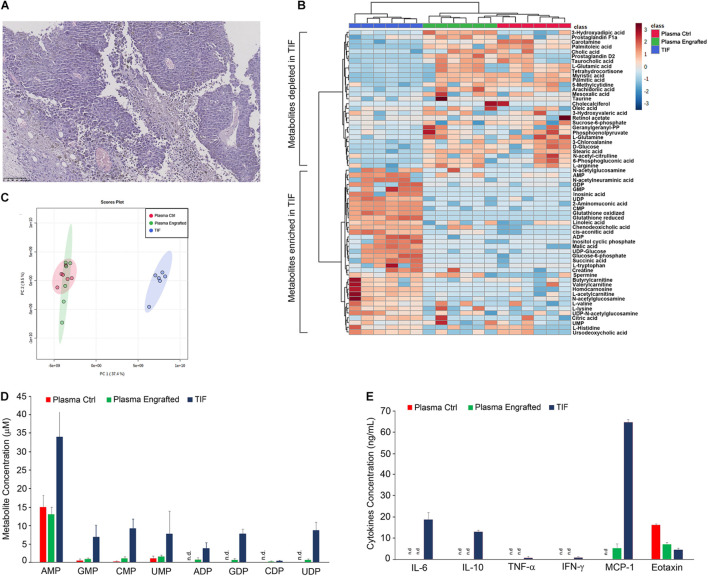
Characterization of melanoma tumor interstitial fluid. **(A)** Histological analysis of tumor, deriving from C57BL6 mice engrafted with 2.0 × 10^5^ B16F10 melanoma cells, stained by hematoxylin and eosin. Original magnification × 10. Hierarchical clustering **(B)** and principal component analysis **(C)** of TIF and plasma samples of tumor and non-tumor-bearing C57BL6 mice, based on LC-MS measurements. **(D)** Quantification by LC-MS of concentration of indicated metabolites in TIF and plasma samples of tumor and non-tumor-bearing C57BL6 mice. Values (mean ± SE, *n* = 6 for each group of samples) are shown. **(E)** Aliquots of TIF and plasma samples (30 μl) of tumor and non-tumor-bearing C57BL6 mice were analyzed for the indicated cytokines and chemokines protein expression. Values (mean ± SE, *n* = 6 for each group of samples) are shown. The asterisk indicates a statistically significant difference according to Student’s *t*-test (*p* < 0.01).

Firstly, we inoculated subcutaneously 2.0 × 10^5^ B16F10 melanoma cells into 2-month-old female C57BL6 mice and, 21 days later, collected melanoma tumors as well as blood samples from the heart, through cardiac puncture upon euthanasia. The microscopic evaluation of H&E-stained sections of tumors showed the presence of large areas of necrotic tissue intermingled with viable tumor cells and numerous congested blood vessels (Figure 1A).

In order to collect melanoma TIF, tumors were also placed on a fine mesh and subjected to low-speed centrifugation, according to the procedure of Haslene-Hox (Haslene-Hox et al., 2011). Metabolites within TIF as well as in plasma of tumor and non-tumor-bearing C57BL6 mice were profiled and quantified by liquid chromatography-mass spectrometry (LC-MS) (Figures 1B–D). Melanoma TIF and plasma samples have been profiled using the described metabolomics techniques and grouped by either hierarchical clustering (Figure 1B) or principal component analysis (PCA, Figure 1C) of metabolite concentrations (Sullivan et al., 2019). Notably, regardless of the methodology applied, the melanoma TIF samples clustered separately from the plasma samples, suggesting that the metabolic composition of melanoma interstitial fluid differs from that of plasma. In addition, no differences were observed in plasma samples from wild type and melanoma tumor-bearing mice (Figures 1B,C). By means of metabolite profiling, we found a statistically significant enrichment (or reduction) of several metabolites in TIF, compared to plasma samples of wild type and tumor-bearing mice (Figure 1B). Among the classes of metabolites analyzed, monophosphate and diphosphate nucleotides resulted enriched in TIF compared to plasma samples (Figure 1D).

We also evaluated the protein amount of a set of 12 cytokines and chemokines, including IL-2, IL-4, IL-5, IL-6, IL-10, IL-12, IL-17A, TNF-α, IFN-γ, MCP-1, MIP-1α, and eotaxin in TIF and plasma samples of C57BL6 mice, engrafted or not engrafted with B16 melanoma cells. Notably, IL-2, IL-4, IL-5, IL-12, IL-17A, and MIP-1α were not detected in any sample. IL-6, IL-10, TNF-α, and IFN-γ were detected only in TIF, although the latter two to a much lesser extent, about 10-fold less (Figure 1E). Interestingly, MCP-1 was detected only in tumor samples and to a much greater extent in TIF, about 10-fold more (Figure 1E). Finally, eotaxin was detected in plasma samples of healthy mice and to a lesser extent in plasma and in TIF of tumor-bearing mice, twofold and fourfold less, respectively (Figure 1E).

### Guanosine Diphosphate and Uridine Diphosphate Modulate Immune Response of CD4^+^ and CD8^+^ T-Cells

Since GDP and UDP were the most enriched metabolites and AMP was the one displaying the highest concentration in TIF, even though it was also present in plasma samples (Figure 1D), we asked whether these metabolites could play a role in modulating the immune response of human PBMCs. Firstly, we tested whether the direct stimulation of PBMCs with AMP, GDP, or UDP could change the composition of some immune subsets, including T-regs.

**FIGURE 2:**
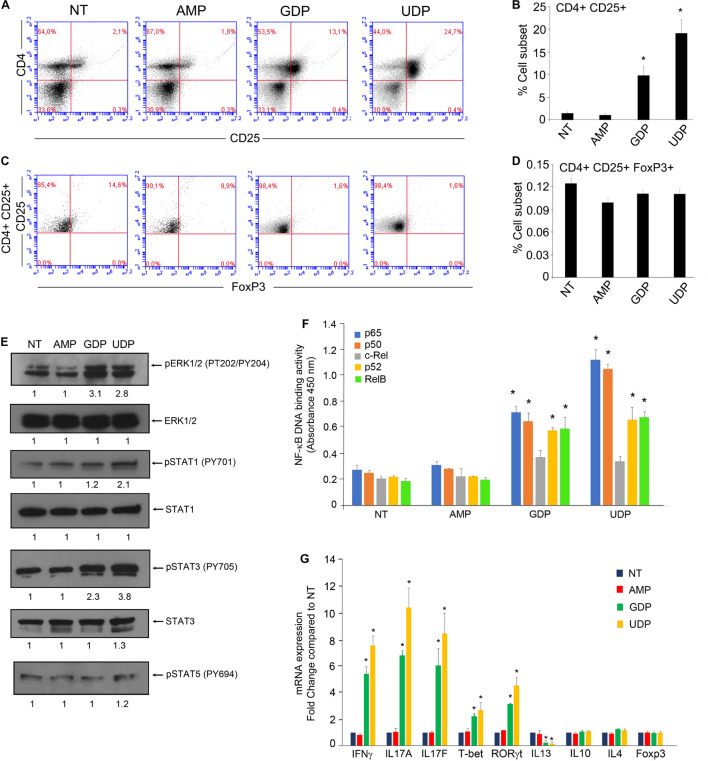
GDP and UDP modulate immune response of CD4^+^ T-cells. **(A)** PBMCs (1 × 10^6^) were treated with 30 μM of indicated metabolites for 24 h and were stained with CD4-FITC and CD25-PE. Each plot represents 20,000 events of a representative experiment. **(B)** Percentage of CD4^+^CD25^+^ population in the different conditions. Values (mean ± SE, *n* = 6) are shown. The asterisk indicates a statistically significant difference compared to untreated control, according to Student’s *t*-test (*p* < 0.01). **(C)** PBMCs stained with CD4-FITC and CD25-PE were permeabilized and next stained with FoxP3-APC. Each plot represents 20,000 events of a representative experiment. **(D)** Percentage of CD4^+^CD25^+^FoxP3^+^ population in the different conditions. Values (mean ± SE, *n* = 6) are shown. The asterisk indicates a statistically significant difference compared to untreated control, according to Student’s *t*-test (*p* < 0.01). **(E)** CD4^+^ T-cells, isolated from PBMCs of healthy donors (5 × 10^6^), were treated with 30 μM of indicated metabolites for 2 h and then were lysed. Total protein extracts (30 μg) were separated by 4–12% gradient NuPAGE and analyzed by Western blotting with anti-pERK1/2 (phospho-tyrosine 202/204), anti-ERK1/2, anti-pSTAT1 (phospho-tyrosine 701), anti-STAT1, anti-pSTAT3 (phospho-tyrosine 705), anti-STAT3, and anti-pSTAT5 (phospho-tyrosine 694). **(F)** CD4^+^ T-cells, isolated from PBMCs of healthy donors (5 × 10^6^), were treated with 30 μM of indicated metabolites for 2 h, and then nuclear extracts were collected. Nuclear extracts (10 μg) were analyzed for the p65, p50, c-Rel, and RelB binding to the NF-κB double-stranded oligonucleotide using the NF-κB Transcription Factor ELISA Assay kit (Cayman). Values are the mean ± SD of three independent experiments. The asterisk indicates a statistically significant difference compared to untreated control, according to Student’s *t*-test (*p* < 0.01). **(G)** PBMCs of healthy donors (5 × 10^6^) were treated with 30 μM of indicated metabolites for 12 h, and then CD4^+^ T-cells were isolated by using CD4^+^ T-cells isolation kit (Miltenyi Biotec). Total RNA was extracted and analyzed by RT-qPCR to evaluate the mRNA expression of the indicated genes. Values are the mean ± SD of three independent experiments. The asterisk indicates a statistically significant difference compared to untreated control, according to Student’s *t*-test (*p* < 0.01).

To this end, we treated PBMCs isolated from buffy coats of healthy donors with 30 μM of indicated metabolites for 24 h and analyzed the percentage of activated CD4^+^ T-cells (CD4^+^CD25^+^) (Figures 2A,B) and T-regs (CD4^+^CD25^+^FoxP3^+^) (Figures 2C,D).

We observed an increase in the CD4^+^CD25^+^ sub-population in PBMCs upon treatment with either GDP or UDP, whereas the AMP treatment was ineffective in this regard. In particular, the CD4^+^CD25^+^ sub-population increased from about 1% to above 10% upon GDP treatment and increased up to 25% upon UDP treatment (Figures 2A,B). Interestingly, none of the nucleotide treatments showed an effect on T-reg sub-population (CD4^+^CD25^+^FoxP3^+^) (Figures 2C,D), whose percentage was about 0.1%.

Since we found an increase in CD25 expression on different immune subsets that compose the IL-2 receptor, whose transcription relies on the activity of the NF-κB pathway (Fu et al., 2013; Grinberg-Bleyer et al., 2018), we asked whether AMP, GDP, and UDP could modulate signaling pathways involved in the immune response of CD4^+^ cells.

In order to check this possibility, we treated for 2 h CD4^+^ T-cells isolated from PBMCs with AMP, GDP, or UDP at a concentration of 30 μM. Compared to control cells, we found that UDP induced ERK phosphorylation at threonine 202 and tyrosine 204, STAT1 at tyrosine 701, and STAT3 at tyrosine 705, while the GDP treatment caused only the increase in ERK and STAT3 phosphorylation. Interestingly, the phosphorylation of STAT5 remained unchanged (Figure 2E). Moreover, by measuring the NF-κB binding activity of nuclear extracts derived from PBMCs subjected to the same experimental conditions, we found that GDP and UDP increased the binding activity of NF-κB subunits p65, p50, RelB, and p52 (Figure 2F).

Following the observations about the activation of critical modulators of immune response including NF-κB family transcriptional factors, STAT1, and STAT3, we asked whether selected nucleotides could regulate the expression of genes encoding for immune response markers. In particular, we analyzed by RT-qPCR the expression of several markers of T-cells immune response, including IFNγ, IL17A, IL17F, IL4, IL13, IL10, T-bet, RORγt, and FoxP3 in CD4^+^ cells isolated from PBMCs, treated for 12 h with the indicated nucleotides at concentration of 30 μM, compared to untreated cells.

We found a significant increase in the expression of mRNAs encoding for IFNΓ, IL17A, IL17F, T-bet, and RORγt and a decrease in expression of IL13, upon both GDP and UDP treatments. On the other hand, the expression of IL4, IL10, and FoxP3 mRNAs remained unaffected (Figure 2G).

Further, we tested whether the stimulation of PBMCs with AMP, GDP, or UDP could affect the proliferation of CD8^+^ T-cells. To this end, CD8^+^ T-cells proliferation was assessed by flow cytometry by measuring the levels of the proliferative marker CFSE (5,6-carboxyfluorescein diacetate, succinimidyl ester, Thermo Scientific) to monitor different generations of proliferating cells due to tracer dilution. PBMCs isolated from a healthy donor (3 × 10^6^) were stimulated with 30 μM of indicated metabolites and covalently linked to the tracer and stimulated. After 3 days, cells were harvested and stained with an anti-CD8-APC conjugated and analyzed by flow cytometry. Following GDP and UDP stimulation, we found an increase in CD8 lymphocyte proliferation that reached 27%, as opposed to the 15% of AMP-treated and unstimulated cells (Supplementary Figures 1A,B). Moreover, we asked whether selected nucleotides could modulate the expression of genes encoding for CD8 effector markers, including perforin, granzyme B, KLRG1, CD127, CD62L, and Eomes. To this end, we first stimulated PBMCs with 30 μM of selected nucleotides and then isolated CD8^+^ T-cells. We found a statistically significant increase in the expression of mRNAs encoding for perforin, granzyme B, CD127, and Eomes in response to both GDP and UDP treatments, whereas KLRG1 and CD62L expression remained unaffected (Supplementary Figure 1C). Altogether, these results prompted us to hypothesize that GDP and UDP could be able to modulate immune response mediated by CD4^+^ and CD8^+^ T-cells.

### Local Administration of Uridine Diphosphate Stimulates Anti-tumor Response Limiting the Tumor Growth and Necrosis in B16-Engrafted C57BL6 Mice

In view of the observation that UDP treatments of PBMCs were able to induce the highest expression of IFNγ (Figure 2G), a typical marker of Th1 anti-tumor response (Kanhere et al., 2012; Tibbitt et al., 2019), and to concomitantly reduce the expression of IL13 (Figure 2G), marker of Th2 immune response (Kanhere et al., 2012; Tibbitt et al., 2019), we asked whether UDP could limit the tumor growth through the stimulation of anti-tumor immune response.

**FIGURE 3:**
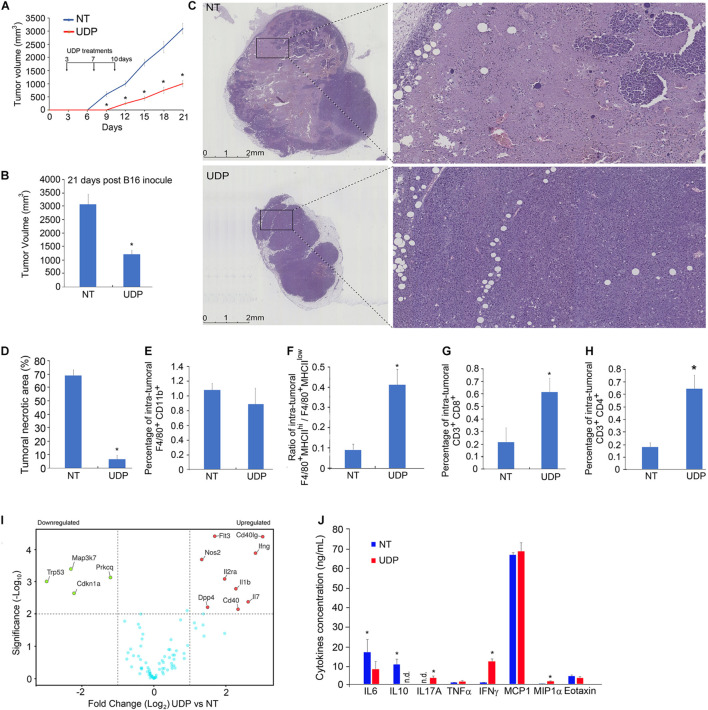
In B16-melanoma-bearing mice, local UDP treatments stimulate anti-tumor response limiting the tumor growth. Two-month-old C57BL6 female mice have been inoculated with 2.0 × 10^5^ B16F10 melanoma cell line and treated, in the same localization of engraftment 3, 10, and 17 days later, with 100 μl of UDP (0.5 mM) or RPMI medium as vehicle. **(A)** Tumor growth curves and **(B)** final volume of tumors explanted 21 days post-engraftment. Values (mean ± SE, *n* = 6 for each group of samples) are shown. The asterisk indicates a statistically significant difference among two groups, according to Student’s *t-*test (*p* < 0.01). **(C)** Histological analysis of tumors treated with vehicle (upper panels) or UDP (lower panels) by hematoxylin and eosin staining at lower (left panels) or higher magnification (right panels). **(D)** Necrosis-to-tumor area ratios comparison among mice implanted with B16 tumors treated with vehicle or UDP. Values (mean ± SE, *n* = 6 for each group of samples) are shown. The asterisk indicates a statistically significant difference among two groups, according to Student’s *t*-test (*p* < 0.01). **(E)** Percentage of intra-tumoral population of F4/80^+^ CD11b + detected by fluorescence-activated cell sorting (FACS) using antibodies F4/80-Percp and CD11b-APC. **(F)** Ratio of F4/80^+^/MHCII^hi^/F4/80^+^/MHCII^low^ intra-tumoral populations detected by flow cytometry using F4/80-Percp and MHCII-FITC antibodies. **(G)** Percentage of intra-tumoral population of TILs CD3^+^CD8^+^ detected by FACS using antibodies CD3-Percp and CD8-FITC. **(H)** Percentage of intra-tumoral population of TILs CD3^+^CD4^+^ detected by FACS using antibodies CD3-Percp and CD4-PE. Values (mean ± SE, *n* = 6 for each group of samples) are shown. The asterisk indicates a statistically significant difference among two groups, according to Student’s *t*-test (*p* < 0.01). **(I)** Total mRNA (1 μg) extracted from UDP- or vehicle-treated tumors of 8-week-old mice was analyzed for expression of 90 genes involved in B- and T-cell activation (RT^2^ Profiler PCR Array B- and T-cell activation mouse, Qiagen). In the volcano plot, genes with a log twofold change ≥ 1 and *p* < 0.01 (*n* = 6 for each group of samples) are considered as differentially expressed. **(J)** Aliquots of TIF (30 μl) isolated from UDP- or vehicle-treated tumors of 8-week-old mice were analyzed for the indicated cytokines and chemokines protein expression. Values (mean ± SE, *n* = 6 for each group of samples) are shown. The asterisk indicates a statistically significant difference according to Student’s *t*-test (*p* < 0.01).

To test this hypothesis, we subcutaneously inoculated 2.0 × 10^5^ cells of B16F10 melanoma line into 2-month-old C57BL6 female mice. After 3, 10, and 17 days, we injected 100 μl of UDP (0.5 mM) or RPMI medium as vehicle, in the same position of tumor engraftments. Twenty-one days post B16-engraftment, mice were sacrificed. Consistent with our previous observations, we found that in mice treated with UDP the tumor growth curve, as well as the final tumor volume, were remarkably reduced, compared to control mice (Figures 3A,B). In addition, the analysis by H&E staining of tumor sections at lower magnification (× 1.25) of control (i.e., vehicle-treated) mice showed how tumors were characterized by large necrotic areas (Figure 3C, top panels, non-treated), whereas UDP-treated mice showed very few and focal necrotic areas (Figure 3C, bottom panels, UDP). In particular, by quantification we reported how the percentage of necrotic areas was above 70% in untreated tumors, whereas it was drastically reduced at less than 5% in UDP-treated tumors (Figure 3D).

The composition analysis of immune infiltrates within tumors, treated with UDP or vehicle, revealed that the percentage of macrophages F4/80^+^ CD11b^+^ was unchanged (Figure 3E). However, the ratio of F4/80^+^MHCII^hi^ population compared to F4/80^+^MHCII^low^ was increased in UDP-treated tumors (Figure 3F), thus suggesting a reduced presence of TAM, with immunosuppressive functions (F4/80^+^MHCII^low^) and a higher presence of macrophages with enhanced antitumor immunity (F4/80^+^MHCII^hi^) (Wang et al., 2011).

In addition, the number of intra-tumoral CD3^+^CD8^+^ and CD3^+^CD4^+^ was increased in UDP-treated tumors compared to control tumors, from 0.2 to 0.6% for CD3^+^CD8^+^ (Figure 3G), and from 0.15 to 0.65% for CD3^+^CD4^+^ (Figure 3H).

To further characterize the anti-tumoral immune response elicited by UDP, we analyze the intra-tumoral mRNA expression profile of a set of 90 genes involved in immune response (Figure 3I) in UDP-treated tumors compared to control, as well as the release of specific cytokines in TIFs (Figure 3J). Strikingly, in UDP-treated tumors comparison, we found an increase in the expression of genes known to be positively involved in T-cell-mediated anti-tumor response, including *Flt3*, *Cd40lg*, *Ifng*, *Nos2*, *Il2ra*, *Il1b*, *Dpp4*, *Cd40*, and *Il7.* Conversely, we found a decrease in the expression of genes such as *Trp53*, *Map3k7*, *Prkcq*, and *Cdkn1a* (Figure 3I).

Moreover, the analysis of specific cytokines released into the interstitial fluids of tumors revealed an increase in cytokines such as IL6, IL17A, IFNγ, and MIP1α and a reduction of IL10 in the interstitial fluids of UDP-treated tumors compared to control (Figure 3J). Taken together, these data indicate that local UDP administration of mice bearing B16 melanoma tumor is able to elicit an immune response exerting an anti-tumoral effect.

## Discussion

TIF represents a major component of tumor microenvironment, containing metabolites and proteins released by tumor and stromal cells or deriving from tumor necrotic areas (Haslene-Hox et al., 2011; Baronzio et al., 2012; Gromov and Gromova, 2016; Lyssiotis and Kimmelman, 2017; Sullivan et al., 2019). In our work, we firstly addressed the questions whether any difference in the concentration of metabolites could be found in the plasma of tumor-bearing as opposed to non-tumor-bearing mice and which were the most enriched or depleted metabolites within melanoma interstitial fluids compared to plasma samples. Both hierarchical clustering and PCA (Figures 1B,C) did not reveal any overt difference in the amount of analyzed metabolites in the plasma of tumor-bearing and non-tumor-bearing mice. Although we were expecting plasma metabolites being differentially expressed among the plasma of non-tumor-bearing versus tumor-bearing mice, we could not find any statistically significant difference between these two datasets. In a recent report, Weber et al. (2021) by using targeted metabolomics have been able to identify seven metabolites differentially expressed in plasma samples of heterogeneous melanoma xenografts, including tiglylcarnitine, beta-alanine, PC ae C42:4, sarcosine, Hex2Cer (d18:1/16:0), Hex2Cer (d18:1/20:0), and p-Cresol sulfate. It is conceivable to speculate that, as a consequence of the different biological settings, our metabolomic profiling did not reveal most of these metabolites, nor, if identified, was it able to determine significant differences in their levels. Nevertheless, melanoma TIF samples clustered separately from the plasma samples, suggesting that the metabolic composition of melanoma interstitial fluid differs from that of plasma (Figures 1B,C). Among the most enriched metabolites in TIFs, we found monophosphate and diphosphate nucleotides and, to lesser extents, some amino acids including histidine, lysine, valine, and tryptophan; Krebs cycle metabolites, such as citric acid, malate, and cis-aconitate; and acyl-carnitines, including butyryl-carnitine and valeryl-carnitine, suggesting a tumor-driven, sustained activity of the Krebs cycle and β-oxidation of fatty acids in mitochondria (Lyssiotis and Kimmelman, 2017; Balaban et al., 2018; Nabi and Le, 2021; Park et al., 2021). Among the most depleted metabolites in TIFs, we found glucose, glutamine, glutamate, and arginine. This is in agreement with several reports indicating that these nutrients represent the main fuel of tumor cells and that their depletion could both induce tumor necrosis and inflammation, limiting TIL activity and causing immunosuppression (Caiazza et al., 2019; Domblides et al., 2019; Gun et al., 2019). In support of this hypothesis, we found large necrotic areas within the tumor (Figure 1A) and high amount of the inflammatory cytokines MCP-1 and IL6 and of immunosuppressive cytokine IL10 (Figure 1E).

In this context, the identification of such a prominent variation in the levels of UDP and GDP was certainly worthy of note. Hence, we asked whether the most enriched metabolites within TIF could modulate immune response, inducing immunosuppression or immune stimulation. To this aim, we firstly treated PBMCs with AMP, GDP, and UDP at a concentration compatible with the one used in previous studies (Li et al., 2014; Neher et al., 2014), which resulted to be entirely safe and non-toxic. Interestingly, GDP and UDP induced dramatic increase in the interleukin-2 (IL-2) receptor (CD25) expression on CD4^+^ cells but not the expression of T-reg marker FoxP3, suggesting a stimulation of T helper response (Hirahara and Nakayama, 2016; Narsale et al., 2019). In support of such an observation, both GDP and UDP were able to increase the expression of mRNA encoding for the cytokines IFNγ, IL17A, IL17F, T-bet, and RORγt gene expression, typical markers of Th1 and Th17 and a reduction of IL13 mRNA expression, marker of Th2 response (Stubbington et al., 2015).

Furthermore, our observation that UDP induced an increase in the phosphorylation status of STAT1 and STAT3 is in agreement with a differentiation toward a Th1 response and Th17 of CD4^+^ cells as the activation of STAT1 is a key factor required for Th1 differentiation (Takeda et al., 2003; Mikhak et al., 2006; Ma et al., 2010), whereas the phosphorylation of STAT3 is essentially required for the induction of Th17 differentiation and for the suppression of T-regs (Yang et al., 2007; Fujino and Li, 2013; Raphael and McGeachy, 2018). Interestingly, we found both GDP and UDP treatment capable of inducing an increase in DNA binding activity of NF-κB subunits p65, p50, RelB, and p52 but not cRel. Despite the fact of NF-κB being essential for both T-cell activation and effector T-cell differentiation, it is also involved in the generation of T-reg cells, mainly through the action of cRel (Ruan et al., 2009).

Consistent with our previous observation, we also found that GDP and UDP induced a slight increase in the proliferation of CD8^+^ T-cells and an increase in expression of mRNA encoding for CD8 effector markers perforin, granzyme B, CD127 and Eomes (Kaech et al., 2002; Pearce et al., 2003; Martin and Badovinac, 2018; Supplementary Figures 1A–C).

As UDP was the strongest inducer of CD4^+^CD25^+^ differentiation, STAT1 and STAT3 phosphorylation, NF-κB activation, and Th1 and Th17 markers expression (IFNγ, IL17A, IL17F, T-bet, and RORγt), as well as the strongest inducer of CD8^+^ T-cell proliferation and of CD8^+^ T-cell effector markers perforin, granzyme B, CD127, and Eomes, it was conceivable to speculate that UDP could trigger an immuno-mediated, anti-tumor response.

To this aim, we treated B16 melanoma cells-engrafted C57BL6 mice with UDP (0.5 mM) by locally administering the tumor engraftment at three different times (3, 10, and 17 days post-engraftment). In agreement with our previous observation, we found that UDP-treated tumors have both volume and necrotic areas considerably reduced compared to control tumors (Figures 3A–D). The number of (F4/80^+^ CD11b^+^) TAMs was similar in both UDP-treated and vehicle-treated tumors (Figure 3E). Nonetheless, the F4/80^+^MHCII^hi^/F4/80^+^MHCII^hi^ ratio was considerably higher in UDP-treated tumors (Figure 3F). It is worth to note that major histocompatibility complex (MHC) class II^hi^ TAM population appears during the early phase of tumor development and is associated with tumor suppression (Wang et al., 2011; Axelrod et al., 2019), whereas the MHC class II^hi^ TAM population is strongly associated with tumor progression, angiogenesis, and metastases formation, since the transition of tumor-associated macrophages from MHC class II^hi^ to MHC class II^low^ mediates tumor progression in mice (Wang et al., 2011; Xiang et al., 2021). Consistent with the hypothesis of UDP acting as an activator of anti-tumor immune response, we also found that in UDP-treated tumors the percentage of TILs CD3^+^CD8^+^ and CD3^+^CD4^+^ were increased (Figures 3G,H). Furthermore, intra-tumoral gene expression analysis of T-cell activation markers showed an increase in the expression of genes functionally linked to anti-tumor immune response, including Flt3 (Bhardwaj et al., 2020; Cueto and Sancho, 2021), Cd40lg (Curran et al., 2015), IFNγ (Gerber et al., 2013), and Il7 (Pellegrini et al., 2009; Figure 3I). Consistent with these observations, we also reported an increase in anti-tumor IFNγ cytokine and a decrease in immunosuppressive IL10 cytokine released within TIF of UDP-treated tumors compared to control (Figure 3J).

From the mechanistic standpoint, we hypothesize that UDP could likely stimulate the anti-tumor response mediated by immune cells by regulating the activity of G-coupled purinergic receptors, which are categorized into three different classes, including P2Y, P2X, and P1 receptors, and are widely expressed in mammalian cells (Elliott et al., 2009; Qin et al., 2016; Li et al., 2018). Among these classes of receptors, P2Y receptors are characterized by ligand selectivity and specificity toward G-protein coupling (Burnstock, 2007). In particular, the P2Y6 receptor has been initially identified as an immune mediator of microglial phagocytosis (Koizumi et al., 2007). In addition, it has been reported how the P2Y6 receptor can be selectively activated by UDP and GDP (Vieira et al., 2011). Interestingly, and in agreement with our observations, in a lung inflammation mice model the P2Y6 receptor signaling was able to stimulate IFNγ production while suppressing the Th2 response (Nagai et al., 2019). Further, UDP treatment could also directly act on tumor cells, reducing their proliferation and, as a consequence, the tumor growth. Indeed, Wan et al. recently showed that the triggering of P2Y6 receptor by UDP treatment in gastric cancer cells caused an increase in Ca^2+^ levels, through store-operated calcium entry (SOCE), and consequently inhibited β-catenin in calcium-dependent manner and cell proliferation, leading to the reduction of tumor growth (Wan et al., 2017).

Hence, it is conceivable to speculate that the dissection of signal transduction pathways downstream of the purinergic receptors in immune and cancer cells will provide additional targets for the development of novel therapeutic strategies. Further work will be needed to address such an interesting possibility.

Arguably, a limitation of our study could be represented by the use of a single melanoma cell line. In order to further corroborate our working model, in a future project we will certainly extend our analysis to other model systems, including additional melanoma cell lines as well as patients-derived human melanoma cells and patients-derived xenograft mice models. However, it is worthy to mention that the availability of samples derived from human melanoma patients is extremely limited. Nevertheless, we will aim at further understanding those indeed interesting and still open questions in a follow-up study.

In conclusion, our analysis revealed UDP as an important metabolite acting as a functional mediator of immune response, which could represent a valuable tool to be used as a co-adjuvant in cancer immunotherapy.

## Data Availability Statement

The raw data supporting the conclusions of this article will be made available by the authors, without undue reservation.

## Ethics Statement

The studies involving human participants were reviewed and approved by the Italian Regional “Calabria” Ethics Committee (Protocol No. 75, 23/03/17). The patients/participants provided their written informed consent to participate in this study. All the experiments involving animals were carried out in accordance with the permission n° 253/2020-PR (prot. 39F3A.61) by the Italian Ministry of Health.

## Author Contributions

GF conceived the project, designed the experimental plan, analyzed and interpreted the data, and wrote the manuscript. EVe, AAl, TB, NN, DM, and MD’A performed gene expression analysis by quantitative real-time PCR and cytokines quantification. CC, SM, and EI performed B16 engraftment in mice. EVi, AI, GD, and CM performed histological analysis. SR and MT performed FACS experiments. FA, GM, and RS performed western blotting analysis. MS performed metabolite profiling and nucleotide quantifications, participated in the interpretation of data, and contributed to the editing of the manuscript. MR edited the manuscript. AAv, AAr, MM, and IQ participated in the analysis of data. All authors contributed to the article and approved the submitted version.

## Conflict of Interest

The authors declare that the research was conducted in the absence of any commercial or financial relationships that could be construed as a potential conflict of interest.

## Publisher’s Note

All claims expressed in this article are solely those of the authors and do not necessarily represent those of their affiliated organizations, or those of the publisher, the editors and the reviewers. Any product that may be evaluated in this article, or claim that may be made by its manufacturer, is not guaranteed or endorsed by the publisher.

## Footnotes

1
https://www.metaboanalyst.ca

2
https://imagej.nih.gov

## Supplementary Material

Supplementary Figure 1 **(A)** PBMCs (3 × 10^6^) were treated with 30 μM of indicated metabolites and then labeled with CellTrace CFSE at a final concentration of 5 μm for 20 min at 37°C. CellTrace CFSE-based proliferation assay was performed on gated CD8^+^ T-cells, by using anti-CD8-APC antibody, *in vitro* cultured for 3 days. **(B)** Percentage of proliferating CD8^+^ T-cells. Values (mean ± SE, *n* = 3) are shown. The asterisk indicates a statistically significant difference compared to untreated control, according to Student’s *t-*test (*p* < 0.01). **(C)** PBMCs from healthy donors (1 × 10^7^) were treated with 30 μM of indicated metabolites for 12 h, and then CD8^+^ T-cells were isolated by using CD8^+^ T-cells isolation kit (Miltenyi Biotec). Total RNA was extracted and analyzed by RT-qPCR to evaluate the mRNA expression of the indicated genes. Values are the mean ± SD of three independent experiments. The asterisk indicates a statistically significant difference compared to untreated control, according to Student’s *t-*test (*p* < 0.01).
