# Supplementary figures and images for: Metabolites Profiling of Melanoma Interstitial Fluids Reveals Uridine Diphosphate as Potent Immune Modulator Capable of Limiting Tumor Growth
Source: Front Cell Dev Biol. 2021 Sep 17;9:730726. doi: 10.3389/fcell.2021.730726 (PMC8486041; doi:10.3389/fcell.2021.730726)

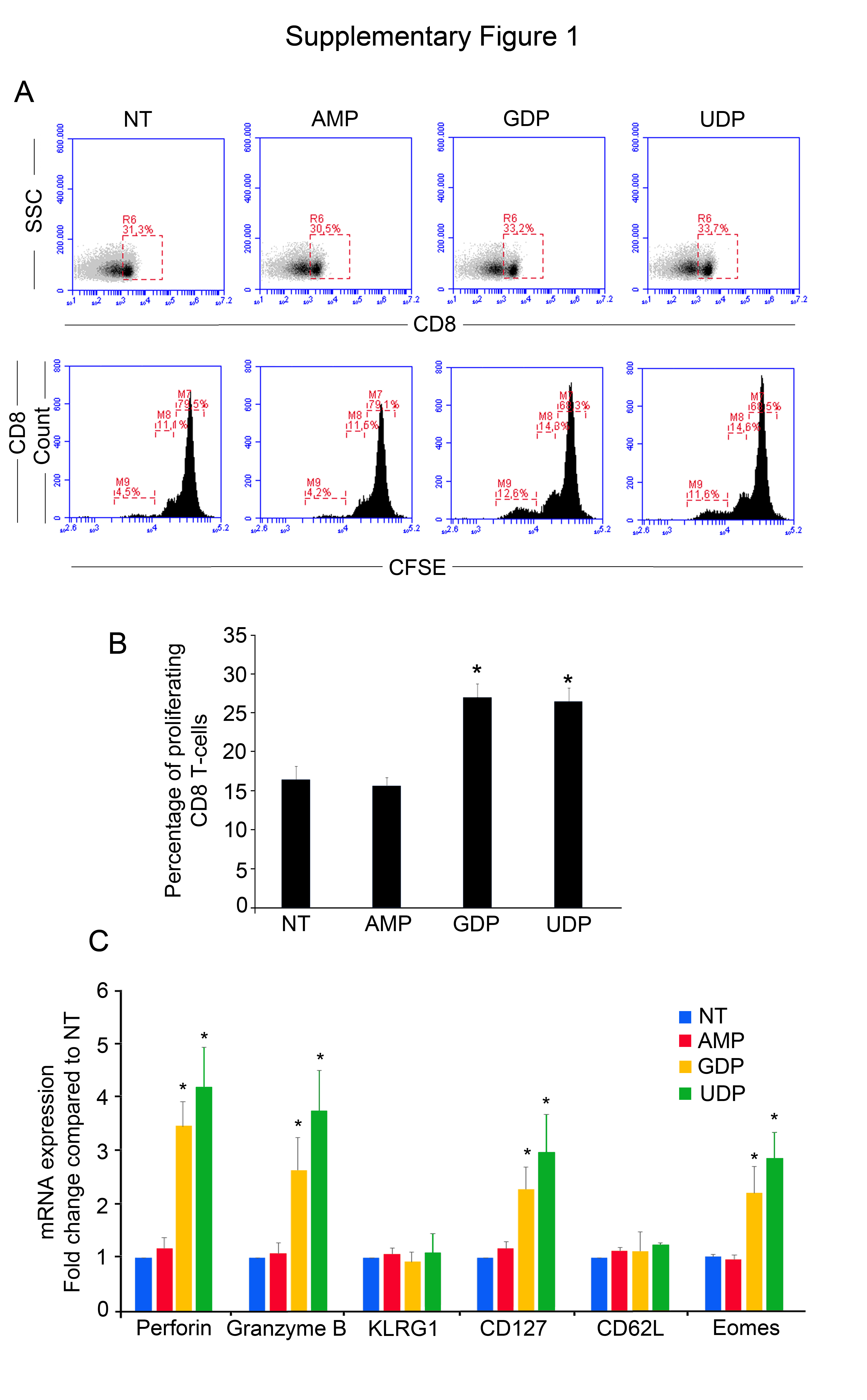

Supplement: Supplementary Figure 1 — (A) PBMCs (3 × 10^6^) were treated with 30 μM of indicated metabolites and then labeled with CellTrace CFSE at a final concentration of 5 μm for 20 min at 37°C. CellTrace CFSE-based proliferation assay was performed on gated CD8+ T-cells, by using anti-CD8-APC antibody, in vitro cultured for 3 days. (B) Percentage of proliferating CD8+ T-cells. Values (mean ± SE, n = 3) are shown. The asterisk indicates a statistically significant difference compared to untreated control, according to Student’s t-test (p < 0.01). (C) PBMCs from healthy donors (1 × 10^7^) were treated with 30 μM of indicated metabolites for 12 h, and then CD8+ T-cells were isolated by using CD8+ T-cells isolation kit (Miltenyi Biotec). Total RNA was extracted and analyzed by RT-qPCR to evaluate the mRNA expression of the indicated genes. Values are the mean ± SD of three independent experiments. The asterisk indicates a statistically significant difference compared to untreated control, according to Student’s t-test (p < 0.01). [file Image_1.jpg]
