# Supplementary material for: Metabolites Profiling of Melanoma Interstitial Fluids Reveals Uridine Diphosphate as Potent Immune Modulator Capable of Limiting Tumor Growth
Source: Front Cell Dev Biol. 2021 Sep 17;9:730726. doi: 10.3389/fcell.2021.730726 (PMC8486041; doi:10.3389/fcell.2021.730726)
Supplement: Supplementary file 2 [file Data_Sheet_1.PDF]

Supplementary Methods

Metabolites profiling of melanoma interstitial fluids reveals UDP as potent immune-modulator capable of limiting tumor growth

Eleonora Vecchio[1], Carmen Caiazza[2], Selena Mimmi[1], Angelica Avagliano[3], Enrico Iaccino[1], Teresa Brusco[2], Nancy Nisticò[1], Domenico Maisano[1], Annamaria Aloisio[1], Ileana Quinto[1], Maurizio Renna[2], Giuseppina Divisato[2], Simona Romano[2], Martina Tufano[2], Massimo D'Agostino[2], Elena Vigliar[3], Antonino Iaccarino[3], Chiara Mignogna[4], Francesco Andreozzi[5], Gaia Chiara Mannino[5], Rosangela Spiga[5], Mariano Stornaiuolo[6], Alessandro Arcucci[3], Massimo Mallardo[2,#] and Giuseppe Fiume[1,#,*]

[1] Department of Experimental and Clinical Medicine, University of Catanzaro 'Magna Graecia', Catanzaro 88100, Italy.

[2] Department of Molecular Medicine and Medical Biotechnology, University of Naples Federico II, Naples 80131, Italy.

[3] Department of Public Health, University of Naples Federico II, Naples 80131, Italy.

[4] Department of Health Sciences, Magna Graecia University, Catanzaro, Italy.

[5] Department of Medical and Surgical Sciences, University "Magna Graecia" of Catanzaro, Catanzaro, Italy.

[6] Department of Pharmacy, University of Napoli Federico II, 80131, Naples, Italy.

* Correspondence and requests for materials should be addressed to G.F. (email: fiume@unicz.it)

G.F. and M.M. should be considered as co-last authors

Primers list

IFNG – Fw: TTCAGCTCTGGATCGTTTTG; Rev: TCTTTTGGATGCTCTGGTCA

IL17F – Fw: CCAAAAGCCTGAGAGTTGCC; Rev: TTGATGCAGCCCAAGTTCCT

FoxP3 – Fw: ACATTTCATGCACCAGCTCTC; Rev: GGGATTTGGGAAGGTGCAGA

RORgT – Fw: GAAGACCCACACCTCACAAA; Rev: CTTGACAGCATCTCGGGACA

IL13 – Fw: CCACGGTCATTGCTCTCACT; Rev: GGCTGCACAGTACATGCCA

IL10 – Fw: GGCACCCAGTCTGAGAACAG; Rev: TGGCAACCCAGGTAACCCTTA

IL4 - Fw: TTGCTGCCTCCAAGAACACA; Rev: TCCAACGTACTCTGGTTGGC

T-bet – Fw: AGGATGTTTGTGGACGTGGT; Rev: TTATGGAGGGACTGGAGCAC

KLRG1 - Fw: TTGCCTACGGCAACCCAAG; Rev: CAGCTGGCACAAGTGGAGTA

CD127 - Fw: CGGGAAGGAGCCAATGACTT; Rev: ATACATTGCTGCCGGTTGGA

Eomes – Fw: AATGGGTGACCTGTGGCAAA; Rev: TTCAAGTCCTCCACGCCATC

Perforin – Fw: CAAGTGCCCCCTGTCTCTG; Rev: TTGTGTGTCCACTGGGAAGG

Granzyme B – Fw: CAGCTGGAGAGAAAGGCCAA; Rev TGGCGTAAGTCAGATTCGCA

CD62L – Fw: GAACTGGGGAGATGGTGAGC; Rev: GCCTTTAGTTTGTGGCAGGC
